# Orthorexia Nervosa: Prevalence Among Spanish University Students and Its Effects on Cardiometabolic Health

**DOI:** 10.3390/nu17040629

**Published:** 2025-02-10

**Authors:** Sara Manero-Higuera, Marta Garcés-Rimón, María Teresa Iglesias-López, Miguel López-Moreno

**Affiliations:** 1 Faculty of Experimental Sciences, Universidad Francisco de Vitoria, 28223 Pozuelo, Spain; saramanero0@gmail.com; 2 Instituto de Investigación en Ciencias de Alimentación, Consejo Superior de Investigaciones Científicas, Universidad Autónoma de Madrid, 28049 Madrid, Spain; marta.garces@ufv.es; 3 Food Biotechnology, Universidad Francisco de Vitoria, 28223 Pozuelo, Spain; 4 Diet, Planetary Health and Performance, Faculty of Health Sciences, Universidad Francisco de Vitoria, 28223 Pozuelo, Spain; miguel.lopez@ufv.es; 5 Physiotherapy, Faculty of Health Sciences, Universidad Francisco de Vitoria, 28223 Pozuelo, Spain

**Keywords:** orthorexia nervosa, prevalence, DOS-ES, stress, university students

## Abstract

**Purpose**: This study aims to determine the prevalence of orthorexia nervosa (ON) among university students and to evaluate the relationship between stress and ON, as well as the effects that ON may have on the health of these individuals. **Methods**: In this cross-sectional study, a total of 205 participants (66.7% women) were recruited through informational posters on the university campus during the 2022–2023 academic year. They answered different questionnaires to yield socio-demographic data and completed specific tests for the evaluation of ON (Düsseldorf Orthorexia Scale (DOS-ES), Eating Habits Questionnaire (EHQ-ES)) and stress (Perceived Stress Scale (PSS-ES)). The analytical determination of blood biomarkers was also carried out. **Results**: The prevalence of ON obtained from the DOS-ES questionnaire was 1.5%, while 7.5% of the individuals showed a risk of ON. In addition, a positive correlation was observed between DOS-ES and EHQ-ES scores (rs = 0.674). A weak correlation (rs = 0.138) was reported between stress and ON. Individuals with underweight BMI (OR: 1.11, 95% CI: 1.01–1.22) and elevated monocyte levels (OR: 1.15, 95% CI: 1.05–1.26) were more likely to have higher DOS-ES scores compared to those with normal weight and normal monocyte levels. **Conclusions**: Our study demonstrated a lower rate (1.5%) than previous studies, and differences by sex or age were not observed in ON diagnosis, nor was a link between underweight BMI and an increased risk of ON. Additionally, a higher monocyte count was associated with ON, suggesting potential immune and cardiometabolic implications, but further research with larger populations is needed to confirm these findings.

## 1. Introduction

Adherence to a healthy lifestyle is essential for the maintenance of optimal health. The term ‘orthorexia nervosa’ (ON), derived from the Greek words *ortho* (right) and *orexi* (appetite), is used to describe a pathological obsession with healthy eating, often characterized by overly restrictive diets and the elimination of certain food groups, which can lead to negative physical and psychological consequences [1]. This term was first described in 1997 by Dr. Steven Bratman in an article published in *Yoga* magazine [2] and further developed in a book entitled “Health Food Junkies: Overcoming the Obsession with Healthful Eating” [1]. The main difference between patients with ON and those with other eating disorders, such as anorexia nervosa or bulimia nervosa, is that they focus more on food quality than quantity [3]. Individuals with ON often exhibit an intense preoccupation with “healthy” eating, which can precipitate emotional distress when deviations from their dietary rules occur. This emotional turmoil not only exacerbates compulsive behavior, but also contributes to increased stress, leading to the exclusion of entire food groups. This compulsive behavior has the potential for clinical deterioration due to malnutrition, detriments to social, academic or vocational functions, and a dependence on healthy eating for positive body image, high self-esteem and satisfaction [4].

So far, ON is not considered an eating disorder, as it is not included in the Diagnostic and Statistical Manual of Mental Disorders (DSM-V) or the International Classification of Diseases (ICD-11) [5,6,7]. This situation is mainly attributable to the absence of a consensus regarding the diagnostic criteria for this condition. Most studies conducted to assess the prevalence of ON in the population are based on the use of the Bratman test for orthorexia (BOT) [1] and the ORTO-15 questionnaire [8], along with its variants, which are adapted to fit the language of the country where the study is conducted [9]. However, these tools have raised concerns due to their lack of validation and standardization, as well as the high prevalence of ON they report. These limitations have highlighted the need for more reliable assessment methods, which has led to the development of alternative scales in recent years [4,10,11,12,13,14]. Some of these alternative methods include the Teruel Orthorexia Scale (TOS) [15], the Düsseldorf Orthorexia Scale (DOS) [16] and the Eating Habits Questionnaire (EHQ) [17]. Data on the prevalence of ON show significant variability depending on the tool used for diagnosis and the sample size of the study [9]. According to a comprehensive meta-analysis performed in 2023, which included a total of 30,476 individuals from 18 different countries, the overall proportion of ON symptoms is 27.5% when using the ORTO-15 questionnaire [18]. However, the interpretation of prevalence data is complicated by the absence of a unified assessment methodology, which is susceptible to biases inherent in the questionnaires used and the cut-off points established in each study.

Although the exact cause of the symptoms associated with ON is unknown, it is believed that it may be a multifactorial process involving different circumstances, such as eating practices learned from parents during the formative years, a previous history of parents with eating disorders, personality traits such as perfectionism, and lastly, overweight or obesity. Other indicators related to the occurrence of ON-associated symptoms are preoccupation with being overweight, concerns with appearance, and a history of eating disorders [19]. It should be noted that pressure to conform to social standards and fear of being judged and isolated for not conforming may also exacerbate orthorexia behaviors [20]. Previous research shows that students in health science fields, particularly nutrition and dietetics, are more prone to disordered eating behaviors, which may even precede their studies and be reinforced by academic pressures and societal body image expectations, potentially exacerbating ON symptoms [21,22]. The physiological consequences of ON often resemble those of other eating disorders, resulting from a lack of essential nutrients, malnutrition, and weight loss associated with the restriction of intake of even whole food groups considered as “unhealthy” [23]. This leads to metabolic and functional imbalances in the body caused by the prolonged state of malnutrition [24]. Among the most frequently observed alterations are mineral deficiencies, hypovitaminosis, anemia, osteoporosis, metabolic acidosis and hyponatremia [25]. Chronic stress can not only worsen the physical effects of malnutrition, but it also intensifies psychological comorbidities such as depression and anxiety, thereby creating a cycle that further perpetuates the harmful impacts of ON [26].

The possible consequences of ON, both physiologically and psychologically, need to be studied in depth, as it is a potentially harmful situation for those who suffer from it and can go unnoticed for a long period of time due to the external perception that the person performs these behaviors to improve their health. Despite increasing attention being paid to ON, significant gaps remain in understanding its prevalence and determinants, especially in specific populations such as health students. The main objective of this study was to evaluate the prevalence of ON in Spanish health students and its relationship with different determinants, including gender, age, body composition and physiological changes.

## 2. Materials and Methods

### 2.1. Study Design and Participants

During the 2022–2023 academic year, a cross-sectional observational study was conducted among health students at the Francisco de Vitoria University (Madrid, Spain). The sample size was calculated using G*Power^®^ software (3.1.9.7; Heinrich Heine University of Düsseldorf, Germany), with a medium effect size, 80% statistical power, and a significance level (α) of 0.05. This calculation determined that a minimum of 190 participants would be required for the study. Informative posters describing the characteristics of the study were distributed around the Francisco de Vitoria University campus to recruit participants. The sample was convenience-based, as participants were selected from the general pool of students at the university. Participants were informed of the purpose of the study and written informed consent was obtained from all subjects. The study sample consisted of undergraduate students from different health sciences degrees from all years including medicine, pharmacy, biotechnology, nursing, physiotherapy and psychology. The inclusion criteria for participation in the study were the following: being enrolled in a health science degree and being between 18 and 35 years of age. In addition, the incomplete registration of the reported questionnaires, defined as cases where participants did not initially complete the questionnaire and failed to respond after multiple follow-up attempts, was considered an exclusion criterion. Online questionnaires were used to collect the data of interest, ensuring anonymity and that only responses with complete information were included. The study was conducted following the guidelines established in the Declaration of Helsinki, and all procedures were reviewed and approved by the ethics committee of the Universidad Francisco de Vitoria (19/2022); the date of approval was 22 March 2022.

### 2.2. Questionnaires

First, a comprehensive socio-demographic questionnaire was administered to collect detailed information on the participants. This included gender, age, average academic grade, parental education levels, employment status, and current place of residence. Participants also provided a self-reported medical history, specifying any diagnosed pathologies and current medication use. Dietary habits were assessed through questions about the use of nutritional supplements, average daily water consumption, the frequency of consuming a second piece of fruit per day, and weekly fast food consumption. The physical activity level of the participants was also assessed using the International Physical Activity Questionnaire (IPAQ) [27].

The following validated questionnaires, suitable for the age range of the study population, were administered to participants individually:

- Spanish version of the Perceived Stress Scale (PSS-ES) [28] used to assess the level of stress perceived during the last month. There is a positive correlation between higher scores and perceived stress levels;
- Spanish version of the Düsseldorf Orthorexia Scale (DOS-ES) [29] used to assess orthorexia behavior through ten statements about healthy eating. The scale is based on a 1 to 4 scoring system from ‘this does not apply to me [1]’ to ‘this does apply to me [4]’. The minimum score is 10 and the cut-off point used is a score > 30 as an indicator of ON. Scores between 25 and 29 indicate a high risk for ON;
- Spanish version of the Eating Habits Questionnaire (EHQ-ES) [30] used to provide a multidimensional measure of knowledge about healthy eating, problems associated with healthy eating and positive feelings about healthy eating. Items are rated on a 4-point scale and high scores indicate tendencies toward orthorexia, with the maximum score being 84.

### 2.3. Anthropometric Measurements

Once the questionnaires had been completed, the participants underwent anthropometric measurements and blood evaluations. Anthropometric measurements were taken by a single trained professional using calibrated SECA^®^ 840 and 877 digital scales (SECA Vogel & Halke, Hamburg, Germany) and SECA^®^ 214 and 217 portable stadiometers (SECA Vogel & Halke, Hamburg, Germany). The weight of the students was assessed barefoot and lightly clothed and was measured in kilograms rounded to the nearest 100 g (0.1 kg). Height was measured with the individual fully erect and with feet together, head in the Frankfort plane and arms hanging freely toward the ground, rounded to the nearest millimetre (0.1 cm). From these data, body mass index (BMI) was calculated using the formula weight (kg)/height^2^ (m^2^). Subjects were classified according to BMI as underweight (BMI < 18.5 kg/m^2^), healthy weight (BMI 18.5–24.9 kg/m^2^), overweight (BMI 25–29.9 kg/m^2^) and obese (BMI ≥ 30 kg/m^2^) according to World Health Organisation (WHO) criteria [31].

### 2.4. Blood Parameters

The analytical determination of biomarkers was performed by authorized personnel from a certified clinical laboratory in Madrid, Spain (Megalab S.L.), using a fasting venous blood sample. The following parameters were determined: glucose (mg/dL), hemoglobin (g/dL), total cholesterol (mg/dL), HDL cholesterol (mg/dL), LDL cholesterol (mg/dL), triglycerides (mg/dL), leukocytes (10^3^ cells/mm^3^), segmented neutrophils (10^3^ cells/mm^3^ and %), lymphocytes (10^3^ cells/mm^3^ and %), monocytes (10^3^ cells/mm^3^ and %), eosinophils (10^3^ cells/mm^3^ and %), basophils (10^3^ cells/mm^3^ and %), and vitamin D (ng/mL). The following ratios were also calculated from these data: total cholesterol/HDL cholesterol, HDL cholesterol/LDL cholesterol, neutrophils/lymphocytes, monocytes/lymphocytes (MLR) and monocytes/HDL cholesterol (MHR). These parameters were selected for their relevance in assessing immune function and inflammation, which can be influenced by the restrictive eating behaviors associated with ON [32].

### 2.5. Statistical Analysis

Statistical analysis was performed using the IBM Statistical Package for the Social Sciences 26.0 (SPSS) software. First, a descriptive analysis of all the demographic and clinical variables was performed. We also tested whether or not the data followed a normal distribution using the Kolmogorov–Smirnov test. Student’s *t*-test (parametric data) and Mann–Whitney U test (non-parametric data) were used to study differences between variables when they had only two categories. One-way ANOVA (parametric data) and Kruskal–Wallis test (non-parametric data) with Bonferroni corrections for multiple comparisons were used for variables with three or more categories. Spearman’s correlation analysis was used to assess the relationship between variables, and the correlation results were categorized as follows: “very strong” (correlation coefficient > 0.7), “moderate” (correlation coefficient = 0.7–0.5), “low” (correlation coefficient = 0.5–0.2), “weak” (correlation coefficient = 0.2–0.1) and “non-existent” (correlation coefficient < 0.1) [33]. The chi-square test was used to compare categorical variables. Multivariate logistic regression analysis of the DOS-ES score with general characteristics, as well as anthropometric and blood parameters, was performed. Potential confounders, including exercise (measured through the IPAQ questionnaire), use of nutritional supplements, other medical conditions, and medication use, were also taken into account in the analysis. The results are shown as odds ratio (OR) values and with 95% Confidence Intervals (IC). The level of statistical significance was set at *p* < 0.05.

## 3. Results

**Figure 1:**
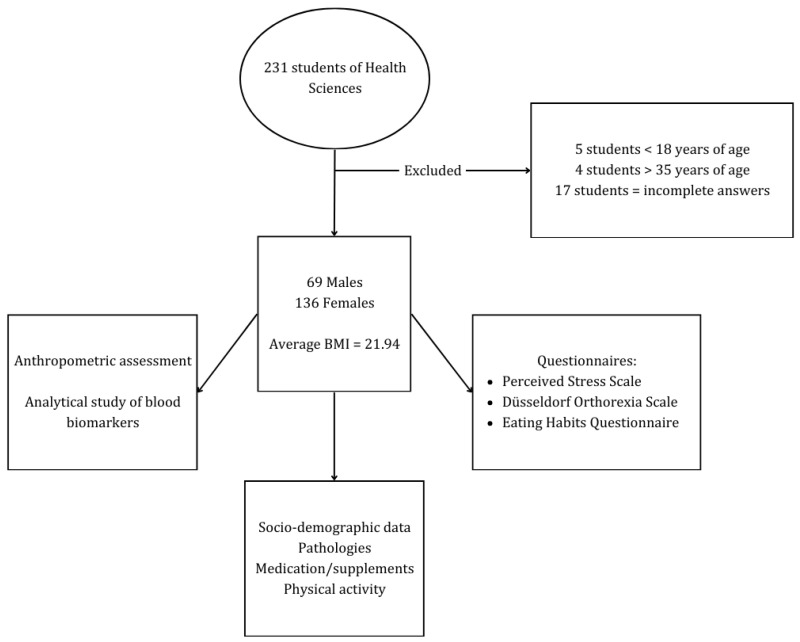
Summary of the participants selected for the study and the process used to obtain the data for analysis.

A total of 231 students participated, of which 9 were excluded for being outside the age range established for the study and 17 more for providing incomplete answers, leaving 205 individuals. A summary of the data collection process is shown in Figure 1.

The proportions of males and females were 33.7% (n = 69) and 66.3% (n = 136), respectively, with an overall mean age of 20.37 years (SD = 3.42). The mean BMI was 21.94 (SD = 3.23). Of the study population, 77.1% had a healthy weight, while 14.6% were overweight or obese. The general characteristics of the study participants are presented in Table 1. Table 1 presents the demographic and lifestyle characteristics of the participants.

The results of the DOS-ES questionnaire indicate that 1.5% (n = 3) of the participants exhibited symptoms consistent with ON, while 7.3% (n = 15) were classified within the ON risk group. The mean scores obtained on the EHQ-ES show significant differences between the groups categorized according to the results of the DOS-ES questionnaire, as follows: 58.33 (SD = 14.29) in individuals with ON symptoms, 54.93 (SD = 6.30) in individuals at risk for ON and 37.28 (SD = 7.20) in individuals without ON (Kruskal–Wallis H = 43.75; *p* < 0.000). Furthermore, when comparing the questionnaire scores directly through Spearman’s correlation, a moderate positive correlation was found between the two variables (rs = 0.674; *p* < 0.000). The Spearman’s correlation analysis results indicate a positive correlation between the PSS-ES and DOS-ES questionnaire scores for the entire study population (rs = 0.138; *p* = 0.048). This correlation was maintained in the men’s group (rs = 0.345, *p* = 0.004), but not in the women’s group.

The results of analytical determinations of blood biomarkers can be found in Table 2. A positive correlation was observed between EHQ-ES and monocyte levels (10^3^ cells/mm^3^) in blood (rs = 0.220, *p* = 0.01) by Spearman correlation in women, but not in men. In addition, a weak positive correlation was observed between EHQ-ES and MLR (rs = 0.178, *p* = 0.038) and MHR (rs = 0.175, *p* = 0.041) in women. Table 2 shows the association between DOS-ES scores and general characteristics and blood parameters. Individuals with below normal BMI showed a higher probability of achieving elevated DOS-ES scores (OR: 1.11, 95% CI: 1.01–1.22) than those with normal weight. Similarly, participants with elevated monocyte levels were more likely to achieve elevated DOS-ES scores (OR: 1.15; 95% CI: 1.05–1.26) compared to those with normal levels.

## 4. Discussion

The main objective of this study was to determine the prevalence of ON among ungraduated health sciences students. The prevalence of ON among this cohort was 1.5%. A weak positive relationship was observed between the DOS-ES and PSS-ES questionnaire scores, as well as a relationship between an increased risk of ON and an underweight BMI. Finally, it was also observed that individuals with higher scores on the DOS-ES questionnaire had a higher blood monocyte count.

Our results obtained using the DOS-ES questionnaire indicate that the prevalence obtained (1.5%) is lower than that previously described in similar study populations in Spain [5,34,35,36]. In a previous study in Spanish university students [34], a discordant prevalence of ON was observed depending on the measurement tool, ranging from 25.5% with the ORTO-11-ES to 10.5% with the DOS-ES. In other countries, also using university populations, a prevalence of less than 1% was reported in the USA using the ORTO-15 questionnaire [37], and 7.8% in China using the DOS questionnaire [38]. These differences could be attributed to sociocultural or socioeconomic factors within the diverse study populations, including variations in cultural contexts when the questionnaire is used in different languages, as well as potential biases arising from the sampling methods and participant characteristics [39]. Moreover, no statistically significant differences in age were observed between individuals with ON or at risk for ON and individuals without ON, suggesting that ON diagnosis is independent of age. Similarly, when comparing the DOS-ES questionnaire scores obtained by males and females, no statistically significant differences were observed between both genders, which is consistent with what has been described so far in the literature [18]. Our findings also suggest that the gender of the individual does not affect his or her probability of suffering from ON.

Another noteworthy finding was the relation between stress reported through the PSS-ES and the score obtained by the DOS-ES questionnaire. The association between the level of stress and the presence of ON symptoms was weak in the general population, while it was low in the men’s group and non-existent in the women’s group. These findings differ from previously described results, as a strong correlation has been identified between the incidence of symptoms compatible with ON and elevated levels of stress in both women and men [40,41,42]. It is possible that this relationship is not observed in this case due to the population sample used in the study. It has already been described that university students in Spain suffer high levels of stress [43], so this may alter the results obtained in the perceived stress questionnaire, with elevated results also being observed in the group of individuals who do not present ON symptoms. Moreover, societal norms can shape how individuals express and cope with stress, with women potentially more likely to externalize or recognize symptoms, while men might internalize stress or exhibit different coping mechanisms [44].

The higher levels of stress reported by participants with higher scores on the DOS-ES questionnaire could have implications for the immune system. In line with this, we found that individuals who presented higher levels of monocytes (10^3^ cells/mm^3^) were more likely to have higher scores on the DOS-ES questionnaire. It has been previously described that psychological illnesses, such as depression or anxiety, are related to the activation of the immune system, especially the innate immune system, increasing circulating monocytes in the blood [32]. It has also been observed that prolonged periods of fasting, as would be the case in people with ON who impose restrictions on the foods they allow themselves to consume and engage in cycles of depurative fasting by breaking their own rules, may adversely affect monocyte function [45]. This altered immune response could contribute to a dysregulated inflammatory environment, potentially increasing the risk and severity of conditions such as ON [46]. Moreover, a weak positive correlation was identified between the MLR and MHR ratios and EHQ-ES scores in women. This could indicate that women with ON symptoms have biomarkers of a chronic inflammatory process that may lead to metabolic disorders such as dyslipidemia, hypertension, atherogenesis and type 2 diabetes mellitus [47]. In addition, an elevated MHR level can be used as a marker for future cardiovascular problems [48,49,50]. A longer follow-up in a larger study population with a greater number of ON cases would be necessary to confirm the relationship between these biomarkers and ON, as well as to better understand the potential implications of these findings on the cardiometabolic health of individuals with ON.

Lastly, our findings indicate that, compared with individuals with normal weight according to the BMI classification, those with below to normal BMI showed a greater likelihood of higher DOS-ES scores. Other research has shown inconsistent evidence on the relationships between ON and BMI. While some describe a negative relationship between BMI and ON [51,52], others have found a positive correlation between both variables [53,54]. Despite these findings, most of the previous research shows that there is no significant relationship between BMI and ON [40,55,56]. The relationship between these two variables appears to depend on the study population used, with a stronger relationship being observed in specific populations such as nutrition students and professionals in this field for example [39]. This suggests that certain populations may exhibit a more pronounced link between BMI and ON due to heightened awareness of eating behaviors and body image. Given that our study population consists of health science students, this may help explain the results we observed. Further research focusing on specific populations could provide valuable insights into the influence of BMI on the risk of ON.

Our study presents several strengths that contribute to the existing literature on orthorexia nervosa and health-related behaviors among university students. First, the use of validated instruments (DOS-ES and EHQ-ES) ensures the reliability and comparability of the findings. Second, the inclusion of a well-defined sample of health science students allows for the exploration of ON in a population that may be at heightened risk due to their educational background and potential preoccupation with nutrition. Additionally, the inclusion of biomarker analysis, particularly blood monocyte levels, provides a novel perspective on the potential biological correlates of ON, thereby offering valuable insights for future research pathways. However, the study also presents several limitations, such as the limited sample size and the fact that it was conducted in a single university center. This may limit the generalizability of our findings to other populations, such as students from other countries or fields of study. The difference in terms of ON diagnostic tools from other studies may also make comparisons difficult. Moreover, as the questionnaires are self-administered by the participants, the reliability of the data provided may be reduced, as the answers are subjective and depend on each individual’s interpretation of the statements. While the questionnaires used have been validated, the possibility of differing personal interpretations means that future research could benefit from employing objective measures alongside self-reports to mitigate these biases [29,30]. Additionally, future studies could explore specific research designs, such as longitudinal or intervention-based studies, to better understand the health consequences of ON and identify effective strategies to reduce the risk of developing this condition. Our findings provide a basis for future studies to shed some light on the possible health problems that ON might cause in these individuals, and the prevalence of ON in university students.

## 5. Conclusions

The prevalence levels of health science students at the Francisco de Vitoria University who present symptoms compatible with ON are 1.5% and 7.3% for those at risk of exhibiting ON according to the DOS-ES questionnaire. No significant differences were observed in terms of gender or age. Moreover, a lower BMI and higher blood monocyte levels were associated with a higher score on the DOS-ES questionnaire, indicating an increased risk of exhibiting ON symptoms. These findings suggest an interconnection between ON and immune system dysregulation, which may contribute to the development of ON symptoms. Given that our findings are based on health science students at a single university, they may not be directly generalizable to other populations. Further large-scale research is needed to explore these associations and their potential health implications in broader populations.

## Figures and Tables

**Table 1:** General characteristics of study participants.

Variables	n (%)/Mean ± SD
**Gender**	
Male	69 (33.7)
Female	136 (66.3)
**Age**	
18–23	182 (88.88)
24–29	15 (7.3)
30–35	8 (3.9)
**Body Mass Index**	
Healthy weight (18.5–24.9 kg/m^2^)	158 (77.1)
Underweight (<18.5 kg/m^2^)	17 (8.3)
Overweight (25–29.9 kg/m^2^)	25 (12.2)
Obesity (≥30 kg/m^2^)	5 (2.4)
**IPAQ**	
Low	91 (44.4)
Moderate	57 (27.8)
High	57 (27.8)
**Use of nutritional supplements**	
Yes	28 (13.7)
No	177 (86.3)
**Pathologies**	
Yes	41 (20)
No	164 (80)
**Use of medication**	
Yes	43 (21)
No	162 (79)
**Score in the Perceived Stress Scale**	25.74 ± 7.70

SD: standard deviation; IPAQ: International Questionnaire of Physical Activity.

**Table 2:** Multivariate logistic regression analysis of DOS-ES scores with general characteristics and blood parameters.

Variables	OR 95% CI	*p*-Value
**Gender**		
Females	Reference	
Males	1.00 (0.94–1.07)	0.93
**Age**		
18–23	Reference	
24–29	1.01 (0.91–1.13)	0.79
30–35	1.13 (0.99–1.28)	0.65
**BMI**		
18.5–24.9	Reference	
<18.5	1.11 (1.01–1.22)	**0.03**
25–29.9	0.98 (0.89–1.08)	0.72
≥30	0.96 (0.77–1.19)	0.70
**Total cholesterol**		
150–199	Reference	
<150	0.98 (0.89–1.08)	0.71
≥200	0.99 (0.93–1.06)	0.86
**HDL-c**		
≥40	Reference	
<40	1.00 (0.92–1.09)	0.95
**LDL-c**		
<130	Reference	
≥130	0.96 (0.83–1.11)	0.60
**TG**		
150–200	Reference	
<150	0.98 (0.88–1.08)	0.70
>200	1.06 (0.93–1.22)	0.39
**Leucocytes**		
4.5–11 × 10^3^ cells/mcL	Reference	
<4.5 × 10^3^ cells/mcL	0.93 (0.76–1.14)	0.50
>11 × 10^3^ cells/mcL	1.13 (0.99–1.28)	0.07
**Monocytes**		
0.14–1.3 × 10^3^ cells/mcL	Reference	
<0.14 × 10^3^ cells/mcL	1.05 (0.84–1.31)	0.66
>1.3 × 10^3^ cells/mcL	1.15 (1.05–1.26)	**0.02**
**Lymphocytes**		
0.77–4.5 × 10^3^ cells/mcL	Reference	
>4.5 × 10^3^ cells/mcL	1.14 (0.93–1.39)	0.22
**Eosinophils**		
0–0.55 × 10^3^ cells/mcL	Reference	
>0.55 × 10^3^ cells/mcL	1.05 (0.89–1.23)	0.56
**Basophils**		
0–0.22 × 10^3^ cells/mcL	Reference	
>0.22 × 10^3^ cells/mcL	1.02 (0.68–1.53)	0.92

BMI: body mass index. LDL-c: low-density lipoprotein cholesterol. HDL-c: high-density lipoprotein cholesterol. TG: triglycerides. Confounders such as diet quality, physical activity (IPAQ), use of nutritional supplements, other medical conditions, and medication use were also accounted for in the model.

## Data Availability

Original data were included into the paper.
